# Identification of Important Amino Acids in Gal2p for Improving the L-arabinose Transport and Metabolism in *Saccharomyces cerevisiae*

**DOI:** 10.3389/fmicb.2017.01391

**Published:** 2017-07-21

**Authors:** Chengqiang Wang, Yanwei Li, Chenxi Qiu, Shihao Wang, Jinjin Ma, Yu Shen, Qingzhu Zhang, Binghai Du, Yanqin Ding, Xiaoming Bao

**Affiliations:** ^1^College of Life Sciences/Shandong Key Laboratory of Agricultural Microbiology, Shandong Agricultural University Tai’an, China; ^2^The State Key Laboratory of Microbial Technology/Environment Research Institute, Shandong University Jinan, China; ^3^College of Bioengineering, Qilu University of Technology Jinan, China

**Keywords:** L-arabinose transport, Gal2p, site-directed mutagenesis, key residue, metabolism, budding yeast

## Abstract

Efficient and cost-effective bioethanol production from lignocellulosic materials requires co-fermentation of the main hydrolyzed sugars, including glucose, xylose, and L-arabinose. *Saccharomyces cerevisiae* is a glucose-fermenting yeast that is traditionally used for ethanol production. Fermentation of L-arabinose is also possible after metabolic engineering. Transport into the cell is the first and rate-limiting step for L-arabinose metabolism. The galactose permease, Gal2p, is a non-specific, endogenous monosaccharide transporter that has been shown to transport L-arabinose. However, Gal2p-mediated transport of L-arabinose occurs at a low efficiency. In this study, homologous modeling and L-arabinose docking were used to predict amino acids in Gal2p that are crucial for L-arabinose transport. Nine amino acid residues in Gal2p were identified and were the focus for site-directed mutagenesis. In the Gal2p transport-deficient chassis cells, the capacity for L-arabinose transport of the different Gal2p mutants was compared by testing growth rates using L-arabinose as the sole carbon source. Almost all the tested mutations affected L-arabinose transport capacity. Among them, F85 is a unique site. The F85S, F85G, F85C, and F85T point mutations significantly increased L-arabinose transport activities, while, the F85E and F85R mutations decreased L-arabinose transport activities compared to the Gal2p-expressing wild-type strain. These results verified F85 as a key residue in L-arabinose transport. The F85S mutation, having the most significant effect, elevated the exponential growth rate by 40%. The F85S mutation also improved xylose transport efficiency and weakened the glucose transport preference. Overall, enhancing the L-arabinose transport capacity further improved the L-arabinose metabolism of engineered *S. cerevisiae*.

## Introduction

Fuel ethanol is an important renewable energy source, and there is a growing demand for the production of this fuel (Farrell et al., 2006; Mabee, 2007). Future large-scale production of fuel ethanol will need lignocellulosic materials, which are renewable and abundant, to replace sugar and grain (Hahn-Hägerdal et al., 2006). Efficient and cost-effective lignocellulosic ethanol production requires co-fermentation of all the main hydrolyzed sugars from lignocellulose, including glucose, xylose, and L-arabinose (Wisselink et al., 2007; Seiboth and Metz, 2011; Wang et al., 2017).

*Saccharomyces cerevisiae* is a traditional ethanol production strain that ferments glucose and could also ferment xylose and L-arabinose by introducing the initial metabolic pathways (Kuyper et al., 2004; Hahn-Hägerdal et al., 2007; Wisselink et al., 2009; Peng et al., 2012; Kim et al., 2013; Wang et al., 2013). However, the consumption of L-arabinose by *S. cerevisiae* is inefficient and inhibited by glucose. Transport into the cell is the first step and one of the rate-limiting steps for L-arabinose utilization, and the transport efficiency needs to be increased (Subtil and Boles, 2012; Shin et al., 2015).

In *S. cerevisiae*, native Gal2p and some heterologous L-arabinose transporters were studied to improve the limiting step of transport. Gal2p (*K*_m_ 57 mM and *V*_max_ 2.2 nmol/min/mg dry mass) was verified as the main L-arabinose transporter; Hxt9p and Hxt10p also exhibited limited L-arabinose transport capacity in some strains (Subtil and Boles, 2011). Some heterologous L-arabinose transporters were also functionally studied in *S. cerevisiae*, such as LAT1p and LAT2p from *Ambrosiozyma monospora*, AraTp (*K*_m_ 3.8 mM and *V*_max_ 0.4 nmol/min/mg dry mass) from *Scheffersomyces stipitis*, Stp2p (*K*_m_ 4.5 mM and *V*_max_ 0.6 nmol/min/mg dry mass) from *Arabidopsis thaliana*, KmAXT1p (*K*_m_ 263 mM and *V*_max_ 57 nmol/min/mg dry mass) from *Kluyveromyces marxianus*, PgAXT1p (*K*_m_ 0.13 mM and *V*_max_ 18 nmol/min/mg dry mass) from *Pichia guilliermondii*, Mgt05860p, Mgt05293p, and Mgt04891p from *Meyerozyma guilliermondii*, LAT-1 (*K*_m_ 58.1 mM and *V*_max_ 116.7 mmol/h/g dry mass) from *Neurospora crassa*, Tct1p from *Trichosporon cutaneum*, Stp1p from *Trichoderma reesei*, and MtLAT-1 (*K*_m_ 29.4 mM and *V*_max_ 10.3 mmol/h/g dry mass) from *Myceliophthora thermophila* (Subtil and Boles, 2011; Verho et al., 2011; Knoshaug et al., 2015; Li et al., 2015; Wang et al., 2015). Although each transporter could transport L-arabinose, transport was inefficient and lower than Gal2p-facilitated transport. Progress has been made in understanding the molecular mechanism of xylose transport (Farwick et al., 2014; Young et al., 2014; Wang et al., 2015); however, the mechanisms involved in L-arabinose transport are largely uncharacterized. As such, future studies are necessary to better understand the complexity and diversity of L-arabinose transporters.

In the present study, we selected the efficient and native L-arabinose transporter, Gal2p, to study the crucial amino acid residues for L-arabinose transport. An L-arabinose transport-deficient chassis was first obtained. The crucial amino acid residues for L-arabinose transport in Gal2p were predicted by XylEp 3D-structure homologous modeling and L-arabinose docking, and then mutated by site-directed mutagenesis. Select mutations in a key residue were determined to be important for L-arabinose transport and were also further evaluated for their impact on glucose and xylose transport.

## Materials and Methods

### Plasmid and Strain Construction

The original *Gal2* fragments were cloned from the genomic DNA of the CEN.PK102-3A yeast strain (Entian and Kötter, 1998) and then inserted into *Eco*R I and *Nco* I sites of the pYX242-*TEF1araA* plasmid (Wang et al., 2013), resulting in the pYX242-*TEF1araA-Gal2* plasmid (the physical map was listed in the **Supplementary Figure S1**). The site-directed mutations of *Gal2* were constructed by a fusion PCR strategy based on the overlap extension PCR (Urban et al., 1997) and then inserted into pYX242-*TEF1araA* using Gibson assembly (Gibson, 2011). The promoter of Gal2p and its mutants was the original promoter *TPI* of plasmid pYX242, and the terminator of them was *PGK1*. *Escherichia coli* DH5α was used for plasmid amplification.

The episomal plasmid pYX2422-*TEF1araA* in a formerly obtained L-arabinose utilizing strain BSW3AP (Wang et al., 2013) was removed to obtain strain BSW4AP (Wang et al., 2017). The *Gal2* gene of BSW4AP was then knocked out by transforming the fusion fragments containing two homologous arms of *Gal2* and a *loxp-KanMX4-loxp* segment cloned from pUG6 (Güldener et al., 1996) to obtain the chassis BSW5AP. Plasmids pYX2422-*TEF1araA*, pYX242-*TEF1araA-Gal2*, and pYX242-*TEF1araA-Gal2m* were transformed into BSW5AP or BSW4EYX (Wang et al., 2015) to test recombinant yeast strains for L-arabinose or xylose and glucose transport. The plasmid YIp5-ara (Wang et al., 2013) was further transformed to BSW4EYX to evaluate the co-utilization and co-fermentation of glucose, xylose, and L-arabinose of Gal2p mutation. Yeast transformations were conducted using the conventional lithium acetate method (Gietz et al., 1995).

*Saccharomyces cerevisiae* strains and plasmids used in this study are listed in **Table 1**. The primers used in this study are summarized in **Table 2**.

**Table 1:** Plasmids and *S. cerevisiae* strains.

Plasmids and strains	Genotype/Properties	Source/Reference
**Plasmids**		
YIp5-ara	YIp5-*HXT7p*-*araA*-*PGK1t*-*HXT7p*-*araB*-*PGK1t*-*HXT7p*-*araD*-*PGK1t*, and selectable marker *loxP-KanMX4-loxP*	Wang et al., 2013
pYX242-*TEF1araA*	pYX242*-PGK1t*-*TEF1p*-*araA*	Wang et al., 2013
pYX242-*TEF1araA-Gal2*	pYX242*-Gal2-PGK1t*-*TEF1p*-*araA*	Present work
pYX242-*TEF1araA-Gal2m*^a^	pYX242*-Gal2m-PGK1t*-*TEF1p*-*araA*	Present work
pUG6	*E. coli* plasmid with segment *loxP-KanMX4-loxP*	Güldener et al., 1996
**Strains**		
CEN.PK102-3A	*MATα leu2-3,112 ura3-52*	Entian and Kötter, 1998
BSW3AP	CEN.PK102-3A derivative; *gre3* (-241, +338):: *TPI1p-RKI1-RKI1t-PGK1p-TAL1-TAL1t-FBA1p-TKL1-TKL1t-ADH1p-RPE1-RPE1t-loxP*, {YIp5-ara, pYX2422-*TEF1araA*}, selected for growth on L-arabinose	Wang et al., 2013
BSW4AP	BSW3AP derivative; discarding plasmid pYX2422-*TEF1araA*	Wang et al., 2017
BSW5AP	BSW4AP derivative; *gal2::KanMX4*	Present work
BSW5AP-A	BSW5AP derivative; {pYX2422-*TEF1araA*}	Present work
BSW5AP-AGal2	BSW5AP derivative; {pYX242-*TEF1araA-Gal2*}	Present work
BSW5AP-AGal2m^b^	BSW5AP derivative; {pYX242-*TEF1araA-Gal2m*}	Present work
BSW4EYX	EBY.VW4000; *rDNA::XYL1-XYL2-XKS1*	Wang et al., 2015
BSW4EYX-Gal2	BSW4EYX derivative; {pYX242-*TEF1araA-Gal2*}	Present work
BSW4EYX-Gal2m^c^	BSW4EYX derivative; {pYX242-*TEF1araA-Gal2m*}	Present work
BSW4EYX-Gal2-A	BSW4EYX derivative; {pYX242-*TEF1araA-Gal2*, YIp5-ara}	Present work
BSW4EYX-F85S-A	BSW4EYX derivative; {pYX242-*TEF1araA-Gal2(F85S)*, YIp5-ara}	Present work

^a^*The pYX242-*TEF1araA*-based plasmids containing all the site-directed mutations of *Gal2* in this work. ^b^The BSW5AP strains containing all the site-directed mutations of *Gal2* expressed in plasmid pYX242-*TEF1araA* in this work. ^c^The BSW4EYX strains containing the selected site-directed mutations of *Gal2* expressed in plasmid pYX242-*TEF1araA* in this work*.

**Table 2:** The DNA oligos used in this work.

Primers	Sequence (5′-3′)	Purpose
Gal2-G418knock-F	ATGGCAGTTGAGGAGAACAATATGCCTGTTGTTTCACAGCA ACCCCAAGCTGGTGAAGACAGCTGAAGCTTCGTACGCTG	Cloning the fragments for *Gal2* deletion
Gal2-G418knock-R	TTATTCTAGCATGGCCTTGTACCACGGTTTGTCGTCATGTTGTAAATCCTCTAAATCGTAGCATAGGCCACTAGTGGATCTG	
Gal2-*Eco*R I-F	CCGGAATTCATGGCAGTTGAGGAGAACAATATGC	Cloning *Gal2*
Gal2-*Nco* I-R	CATGCCATGGTTATTCTAGCATGGCCTTGTAC	
Gal2-One22-F	AAAAAACACATACAGGAATTCATGGCAGTTGAGGAGAACAATATGC	Cloning *Gal2 for one-step clone*
Gal2-One22-R	CCTAGCTAGCTAGATCCATGGTTATTCTAGCATGGCCTTGTAC	
Gal2-F85-F	TTCGGCGGCTTCATGNNNGGCTGGGATACCGGT	Obtaining the F85 site mutations of *Gal2*


Gal2-F85-R	TACCGGTATCCCAGCCNNNCATGAAGCCGCCGAAG	
Gal2-T89-F	CATGTTTGGCTGGGATNNNGGTACTATTTCTGGG	Obtaining the T89 site mutations of *Gal2*


Gal2-T89-R	CCCAGAAATAGTACCNNNATCCCAGCCAAACATG	
Gal2-F223-F	ATTACTGCAGGTATCNNNTTGGGCTACTGTACT	Obtaining the F223 site mutations of *Gal2*


Gal2-F223-R	AGTACAGTAGCCCAANNNGATACCTGCAGTAAT	
Gal2-N3467-F	CAACAATTAACCGGTNNNNNNTATTTTTTCTACTACGG	Obtaining the two sites N346 and N347 mutations of *Gal2*


Gal2-N3467-R	CCGTAGTAGAAAAAATANNNNNNACCGGTTAATTGTTG	
Gal2-F3501-F	CCGGTAACAATTATTTTNNNNNNTACGGTACCGTTATT	Obtaining the two sites F350 and Y351 mutations of *Gal2*


Gal2-F3501-R	AATAACGGTACCGTANNNNNNAAAATAATTGTTACCGG	
Gal2-N376-F	GTCATTGGTGTAGTCNNNTTTGCCTCCACTTTC	Obtaining the N376 site mutations of *Gal2*


Gal2-N376-R	GAAAGTGGAGGCAAANNNGACTACACCAATGAC	
Gal2-Y446-F	CCTGTTTTTATATTTTCTGTNNNGCCACAACCTGGGCG	Obtaining the Y446 site mutations of *Gal2*


Gal2-Y446-R	CGCCCAGGTTGTGGCNNNACAGAAAATATAAAAACAGG	
pYX242-ce-F	GGAGTTTAGTGAACTTGCAAC	Plasmid pYX242 verifying and the *Gal2* mutation sequencing


pYX242-ce-R	CGACTCACTATAGGGCGAATTG	
PGKt-pYX2422-R	ATACGCTGAACCCGAACATAG	

### Media and Batch Cultivation

The yeast synthetic complete (SC) medium used in this study contains 1.7 g L^-1^ yeast nitrogen base (YNB, Sangon, China) and 5 g L^-1^ ammonium sulfate (Sangon, China). To cultivate the yeast and maintain the required plasmids, the medium was supplemented with the appropriate carbon source and complete supplement mixture (i.e., 0.77 g L^-1^ CSM-URA, 0.69 g L^-1^ CSM-LEU, or 0.67 g L^-1^ CSM-LEU-URA) (MP Biomedicals, Solon, OH, United States). For strains containing the KanMX4 marker, the medium was supplied with 200 μg mL^-1^ G418 sulfate (Promega, Madison, WI, United States). Plasmids were amplified in *E. coli* strain DH5α (TransGenBiotech, China), which was grown on Luria-Bertani (LB) medium with 200 μg mL^-1^ ampicillin.

To cultivate BSW3AP-based strains, single colonies were preincubated in SC medium containing 20 g L^-1^ glucose for 24 h, and then shifted into medium containing 5 g L^-1^ glucose and 15 g L^-1^ L-arabinose for 48 h. After that, the cells were collected and used for batch cultivation in the respective SC medium containing 20 g L^-1^ L-arabinose at an initial OD_600_ of 1. To cultivate BSW4EYX-based strains, single colonies were pre-incubated in SC medium containing 20 g L^-1^ maltose for 24 h, and then the cells were collected and used for batch cultivation in the respective SC medium containing 20 g L^-1^ glucose, 20 g L^-1^ xylose, or 20 g L^-1^ L-arabinose at an initial OD_600_ of 1 or 5. All the yeast strains were batch cultivated in 40 mL cultures in 150 mL aerobic triangular flasks or air-limited flasks at 30°C, 200 r min^-1^. All *E. coli* strains were cultured at 37°C, 200 r min^-1^.

### Homologous Modeling of Gal2p for L-arabinose Binding Suggests Crucial Amino Acid Residues

The putative homology model of the transporter Gal2p was generated using Discovery Studio software (DS, Accelrys, San Diego, CA, United States). The *E. coli* homolog of the glucose transporters GLUT1-4 (PDB code 4GBY, 4GBZ, and 4GC0) (Sun et al., 2012) served as template to construct the homology model using the MODELER auto module. The CHARMm-based molecular mechanics was used to refine the loop regions of the protein structure. PROCHECK (Laskowski et al., 1993) was used to validate the refined models. The locations of L-arabinose were determined by core-constrained protein docking and a modified CHARMm-based CDOCKER method. The best position of L-arabinose was chosen by comparing the CDOCKER energy among the determined 10 positions. The critical amino acid residues at the L-arabinose binding position were analyzed using VMD software (Humphrey et al., 1996).

### Site-Directed Mutagenesis of *Gal2* and Large-Scale Screening

To investigate the function of amino acid residues of Gal2p for L-arabinose, xylose, or glucose transport, we utilized site-directed random mutagenesis. The variety of the residues were introduced by the design of primers, with the coded three bases replaced by NNN (Farwick et al., 2014).

The plasmids containing different mutants of *Gal2* were ligated and directly transformed into BSW5AP; then cells were spread onto solid medium with 20 g L^-1^ L-arabinose or 20 g L^-1^ L-arabinose together with 2 g L^-1^ glucose. The colonies that were distinctly larger or smaller than the BSW5AP-AGal2 strain were selected out. The exponential growth rates of the selected colonies were subsequently determined in the secondary screening procedure on 20 g L^-1^ L-arabinose. The plasmids in the selected yeasts were then extracted and sequenced to identify specific mutations.

### Growth Measurement

The culture optical density (OD_600_) was measured by a BioPhotometer plus (Eppendorf, Germany) and was used to determine the strain growth. The growth capacities of strains were determined by the exponential growth rates (Young et al., 2014), which were analyzed by the linear regression coefficients of ln OD_600_ versus growth hours from the growth curves (Wang et al., 2015).

### The Analysis of Metabolites

The concentrations of glucose, xylose, xylitol, L-arabinose, arabitol, and ethanol were determined using the supernatant of filtered samples collected from batch cultivation. The high-performance liquid chromatography (HPLC) prominence LC-20A (Shimadzu, Japan), which has a refractive index detector RID-10A (Shimadzu, Japan) and an Aminex HPX-87P ion exchange column (Bio-Rad, United States), was used to determine the concentration of the above chemicals at 80°C with a mobile phase of water at a flow rate of 0.6 mL min^-1^, as previously reported (Garcia Sanchez et al., 2010; Wang et al., 2013).

## Results

### The Construction of L-arabinose Transport-Deficient Chassis

**FIGURE 1:**
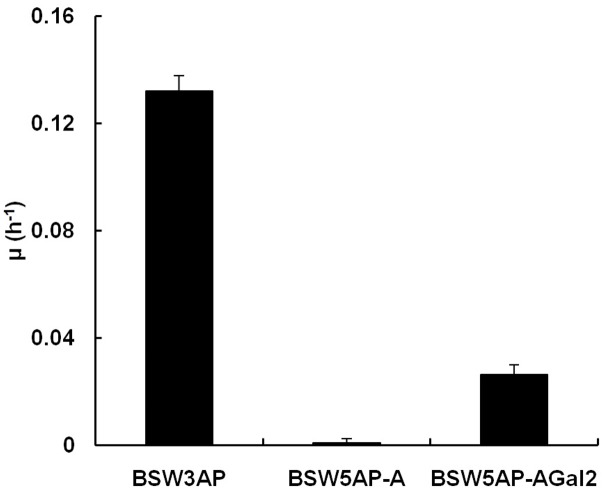
The exponential growth rates of strains BSW3AP, BSW5AP-A, and BSW5AP-AGal2 on 20 g L^-1^ L-arabinose. The strains were preincubated and then collected for aerobic batch cultivation in 40 mL SC-Leu-Ura medium with 20 g L^-1^ L-arabinose at 30°C, 200 r min^-1^. The initial OD_600_ was 1. The data presented are the averages of three independent tests.

To construct L-arabinose transport-deficient chassis and study the L-arabinose transport mechanism, the endogenous and highly efficient L-arabinose transporter *Gal2* of BSW4AP was knocked out using the *KanMX4* marker to generate the strain BSW5AP. To test the L-arabinose transport capacity of BSW5AP, plasmids pYX2422-*TEF1araA* and pYX2422-*TEF1araA-Gal2* were then transformed to generate the strains BSW5AP-A and BSW5AP-AGal2, respectively. The growth curves of the strains BSW5AP-A, BSW5AP-AGal2, and BSW3AP on L-arabinose were tested, and the exponential growth rates were calculated (**Figure 1**). It was clear that BSW5AP-A lost the ability to grow on L-arabinose. After overexpressing *Gal2* by plasmid in BSW5AP, the BSW5AP-AGal2 strain regained the ability to grow on L-arabinose; however, the exponential growth rate of BSW5AP-AGal2 was lower than BSW3AP, which may have resulted from the different expression strategy. We verified that our strains exhibited Gal2p-dependent L-arabinose transport, and we successfully generated the L-arabinose transport-deficient chassis BSW5AP.

### Searching for Functional Amino Acid Residues of Gal2p in L-arabinose Transport by Homologous Modeling

**FIGURE 2:**
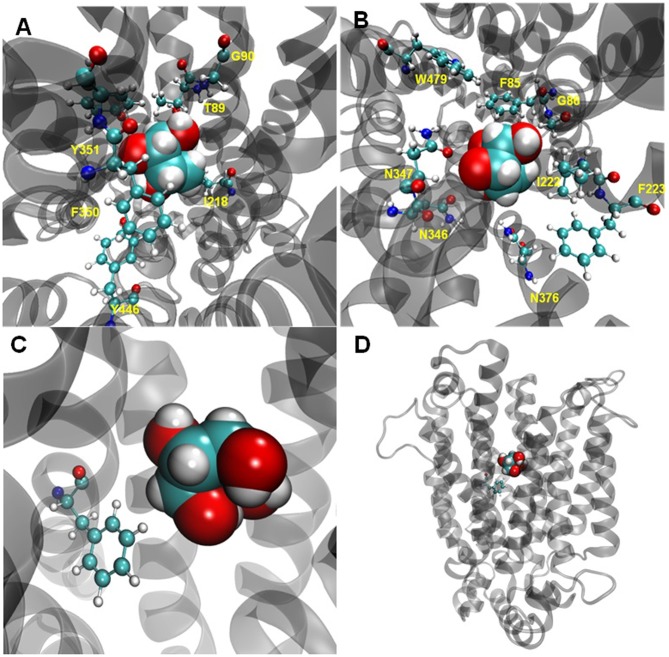
The homologous modeling and crucial amino acid residues identified in Gal2p to be important for L-arabinose binding. The 14 amino acid residues within a distance of 5 Å to L-arabinose in the model **(A,B)**. The position of F85 site with L-arabinose in the model **(C,D)**. The homology model of Gal2p with L-arabinose was generated according to the outward-facing and partly occluded 3D structure of XylEp using the Discovery Studio software. The middle of the Gal2p model placed a colored L-arabinose using the space-filling model. The predicted amino acid residues are also colorfully presented around L-arabinose using the ball-and-stick model.

The homologous model of Gal2p for L-arabinose binding was constructed according to the outward-facing and partly occluded conformation of the *E. coli* xylose permease XylEp (Sun et al., 2012). The homology of Gal2p with XylEp was more than 28.8%, which indicated the feasibility to construct a homologous model. The homologous model of Gal2p was constructed by excluding 63 amino acids from the N-terminus and 39 amino acids from the C-terminus due to the absence of the corresponding sequences in XylEp. A total of 14 amino acid residues within a distance of 5 Å to L-arabinose in the model were predicted (**Figures 2A,B** and **Table 3**). The polar or aromatic amino acid residues in different transmembrane sequences (TMSs) of Gal2p were chosen for more in-depth study, including F85, T89, F223, N346, N347, F350, Y351, N376, and Y446.

**Table 3:** The amino acid residues identified by homologous modeling of Gal2p for L-arabinose binding.

The amino acid residues within 5 Å distance with L-arabinose	The position of the amino acid residues	Within 3 Å distance with L-arabinose	Hydrogen bonding with L-arabinose
F85	TMS1	Yes	No
G86	TMS1	No	No
T89	TMS1	Yes	No
G90	TMS1	No	No
I218	TMS5	Yes	No
I222	TMS5	Yes	No
F223	TMS5	No	No
N346	TMS7	Yes	Yes
N347	TMS7	Yes	Yes
F350	TMS7	Yes	No
Y351	TMS7	Yes	No
N376	TMS8	No	No
Y446	TMS10	No	No
W479	TMS11	No	No

### The Demonstration of Crucial Amino Acid Residues for L-arabinose Transport in Gal2p

To investigate the function of the selected amino acid residues of Gal2p in either L-arabinose, xylose, or glucose transport, we used random and site-directed mutagenesis to introduce amino acid substitutions. The exponential growth rates of the expressing BSW5AP strains during aerobic cultivation were used to determine the change in growth capacity (**Table 4**).

**Table 4:** The effect of the amino acid substitutions in Gal2p on L-arabinose transport.

Transporter or mutation sites	The position of the mutation site	Exponential growth rates (μ) on L-arabinose (h^-1^)	FC_L-arabinose_^a^
Control		0.001 ± 0.000	0.040


Gal2p		0.025 ± 0.001	1.000


F85G	TMS1	0.033 ± 0.001	1.320


F85S	TMS1	0.035 ± 0.001	1.400


F85Y	TMS1	0.026 ± 0.002	1.040


F85C	TMS1	0.030 ± 0.001	1.200


F85T	TMS1	0.029 ± 0.001	1.160


F85N	TMS1	0.026 ± 0.000	1.040


F85L	TMS1	0.026 ± 0.003	1.040


F85V	TMS1	0.025 ± 0.001	1.000


F85E	TMS1	0.001 ± 0.001	0.040


F85R	TMS1	0.001 ± 0.001	0.040


T89H	TMS1	0.001 ± 0.000	0.040


T89K	TMS1	0.002 ± 0.001	0.080


T89R	TMS1	0.001 ± 0.001	0.040


T89N	TMS1	0.003 ± 0.001	0.120


T89P	TMS1	0.024 ± 0.000	0.960


T89I	TMS1	0.027 ± 0.001	1.080


T89Y	TMS1	0.005 ± 0.001	0.200


T89G	TMS1	0.009 ± 0.000	0.360


F223R	TMS5	0.001 ± 0.001	0.040


F223E	TMS5	0.011 ± 0.002	0.440


F223C	TMS5	0.028 ± 0.001	1.120


F223L	TMS5	0.027 ± 0.002	1.080


F223Q	TMS5	0.029 ± 0.001	1.160


F223S	TMS5	0.027 ± 0.001	1.080


N346AN347G	TMS7	0.021 ± 0.002	0.846


F350AY351A	TMS7	0.001 ± 0.000	0.038


F350AY351H	TMS7	0.004 ± 0.002	0.160


F350AY351N	TMS7	0.000 ± 0.000	0.012


F350AY351V	TMS7	0.002 ± 0.000	0.064


F350GY351Q	TMS7	0.001 ± 0.000	0.054


F350PY351F	TMS7	0.001 ± 0.001	0.048


N376S	TMS8	0.019 ± 0.001	0.760


N376T	TMS8	0.021 ± 0.001	0.840


N376Y	TMS8	0.000 ± 0.000	0.000


N376G	TMS8	0.021 ± 0.004	0.840


N376K	TMS8	0.000 ± 0.000	0.000


N376R	TMS8	0.001 ± 0.000	0.040


N376F	TMS8	0.009 ± 0.002	0.360


N376C	TMS8	0.021 ± 0.001	0.840


N376A	TMS8	0.018 ± 0.006	0.720


N376I	TMS8	0.008 ± 0.001	0.320


Y446A	TMS10	0.000 ± 0.000	0.000


Y446I	TMS10	0.001 ± 0.000	0.040


Y446C	TMS10	0.001 ± 0.000	0.040


Y446S	TMS10	0.001 ± 0.000	0.040

^a^*The fold change of exponential growth rates on L-arabinose (μ_*mutants*_/μ_*Gal2p*_)*.

A total of 10 substitution mutants were identified at the selected F85 site. When F85 was changed to a charged E or R, Gal2p lost the transport capacity of L-arabinose. However, changes to the polar amino acids S, G, C, T, Y, or N of F85 somehow increased the exponential growth rates. Furthermore, the exponential growth rates of the F85S, F85G, F85C, and F85T mutants on L-arabinose significantly increased by 40, 32, 20, and 16%, respectively, compared with the strain with wild-type Gal2p. As such, F85 was identified as a very important amino acid residue for L-arabinose transport (**Figures 2C,D**). For site T89, except for the slight increase in exponential growth when changed to an I residue, changes to H, R, and K all resulted in a decrease or loss of growth capacity on L-arabinose. Another important amino acid residue for L-arabinose transport of Gal2p is F223. When F223 was changed to Q, C, L, and S, the exponential growth rates increased, and F223Q significantly improved by 16%. We changed the amino acids N346 and N347 together and found that, even when changed to small side chain amino acids A and G, respectively, the L-arabinose transport capacity of Gal2p was not remarkably reduced. Random mutation of amino acids F350 and Y351 resulted in a loss in the L-arabinose transport capacity of Gal2p. Mutations to the amino acid residue N376 in TMS 8 of Gal2p also presented complex effects on L-arabinose transport. Changing N376 to Y, R, and K, resulted in loss of Gal2p transport capacity. The mutations N376T, N376G, N376C, N376S, N376A, N376F, and N376I decreased the exponential growth rates by 16, 16, 16, 24, 28, 64, and 68%, respectively. Mutating the Y446 residue to A, I, C, or S resulted in a loss of transport capacity. This result indicated that Y446 is very conservative and is necessary for L-arabinose transport in Gal2p.

### The Impact of F85 Positive Mutations on L-arabinose Metabolism

**FIGURE 3:**
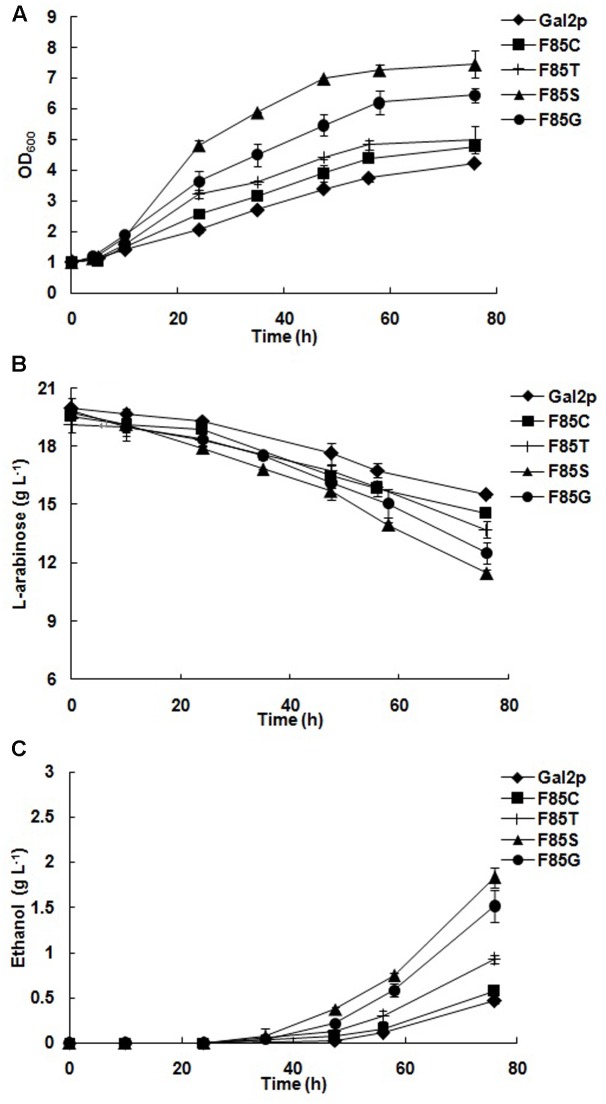
The effect of F85 positive mutants of Gal2p on L-arabinose metabolism. Growth curve **(A)**, L-arabinose consumption **(B)**, and ethanol formation **(C)** by Gal2p (BSW5AP-AGal2, 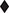), F85C (BSW5AP-AGal2 (F85C), 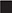), F85T (BSW5AP-AGal2 (F85T), **+**), F85S (BSW5AP-AGal2 (F85S), 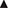), and F85G (BSW5AP-AGal2 (F85G), 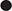). The pre-cultured strains were then cultured in 40 mL SC-LEU-URA medium containing 20 g L^-1^ L-arabinose at an initial OD_600_ of 1. The strains were all cultured at 30∘C, 200 r min^-1^. The data presented are the averages of three independent tests.

The enhanced L-arabinose transport capacity of positive mutations F85S, F85G, F85C, and F85T of Gal2p was further examined by L-arabinose metabolism. The L-arabinose metabolism capacity was evaluated by oxygen-limited batch cultivation. The L-arabinose metabolizing and ethanol producing were deeply concerned. The growth curves and L-arabinose metabolic trend are presented in **Figure 3**.

The L-arabinose metabolic efficiency positively correlated with the exponential growth rates on L-arabinose (**Table 4**). F85S and F85G exhibited rapid growth rates on L-arabinose and utilization of L-arabinose. During the 76 h fermentation, the Gal2p-expressing strain utilized 4.5 g L-arabinose and consumed only 22%. The L-arabinose utilization of F85S and F85G increased to 8.3 and 7.3 g, and the consumption amounts were 42 and 37%, respectively. Compared with the wild-type Gal2p-expressing strain, the consumption of L-arabinose by the F85S and F85G mutants increased by 84 and 62%, respectively. Furthermore, the F85S and F85G mutants produced 1.4 and 1.1 g L^-1^ more ethanol than the wild-type Gal2p-expressing strain.

**FIGURE 4:**
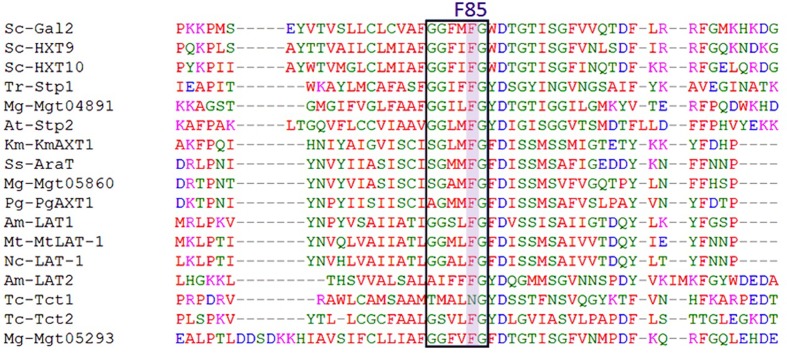
Partial protein sequence alignment of L-arabinose transporters in *S. cerevisiae*, using MUSCLE (3.8) (http://www.ebi.ac.uk/Tools/msa/muscle). Sc, *S. cerevisiae*; Am, *A. monospora*; Ss, *S. stipitis*; At, *A. thaliana*; Km, *K. marxianus*; Pg, *P. guilliermondii*; Mg, *M. guilliermondii*; Nc, *N. crassa*; Tc, *T. cutaneum*; Tr, *T. reesei*; Mt, *M. thermophila*. The sequences in the textbox represent the “G-G/F-XXX-G” motif in the first transmembrane span.

From the results above, we can predict that the change in F85 to polar amino acids will improve the L-arabinose transport capacity of Gal2p. The F85 site of Gal2p is also very conservative in the L-arabinose transporters expressed in *S. cerevisiae* and is located in the “G-G/F-XXX-G” motif in the first transmembrane span (**Figure 4**). The universal meaning of this key site was presented.

### The Impact of the Positive L-arabinose Transport Mutants on Glucose and Xylose Transport

**FIGURE 5:**
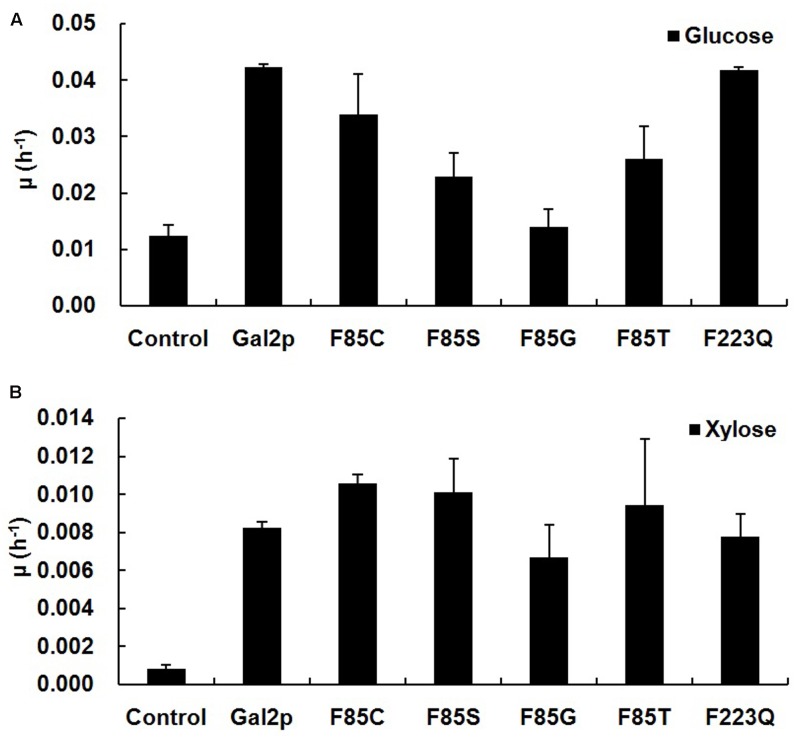
The exponential growth rates of positive L-arabinose transport mutants of Gal2p on glucose **(A)** and xylose **(B)**. The BSW4EYX strain contains the plasmid pYX242-*TEF1araA*, which does not express the Gal2p transporter. The other strains are represented by the transporter Gal2p-expressing wild-type strain or its mutants. The BSW4EYX-based strains were preincubated in SC-LEU medium containing 20 g L^-1^ maltose for 24 h and then collected and used for batch cultivation in 40 mL SC-LEU medium containing 20 g L^-1^ glucose or 20 g L^-1^ xylose at 30°C, 200 r min^-1^ and the initial OD_600_ was 1. The data presented are the averages of three independent tests.

Endogenous Gal2p of *S. cerevisiae* can transport many sugars, including glucose, xylose and L-arabinose (Leandro et al., 2009). To test the glucose and xylose transport capacities of the positive L-arabinose transport mutations, F85S, F85G, F85C, F85T, and F223Q of Gal2p, these mutants were transformed into the BSW4EYX strain, which lost the hexose and pentose transport capacities and contained the XR/XDH pathway of xylose (Wieczorke et al., 1999; Wang et al., 2015). The exponential growth rates of the BSW4EYX expressing strains were also used to determine the change of growth capacity on glucose- (**Figure 5A**) and xylose-containing medium (**Figure 5B**). Unlike mutation F223Q, the F85G, F85S, F85T, and F85C mutations all decreased the glucose transport efficiency by 67, 45, 38, and 19%, respectively. This result indicated that the benzene ring of F85 in Gal2p might be important for glucose transport. For xylose transport of Gal2p, the F85C, F85S, and F85T mutations improved the xylose transport efficiency, and the exponential growth rates increased by 29, 23, and 15%, respectively. However, the exponential growth rate of the F85G mutant decreased by 19%, and F223Q did not change the growth efficiency on xylose.

**FIGURE 6:**
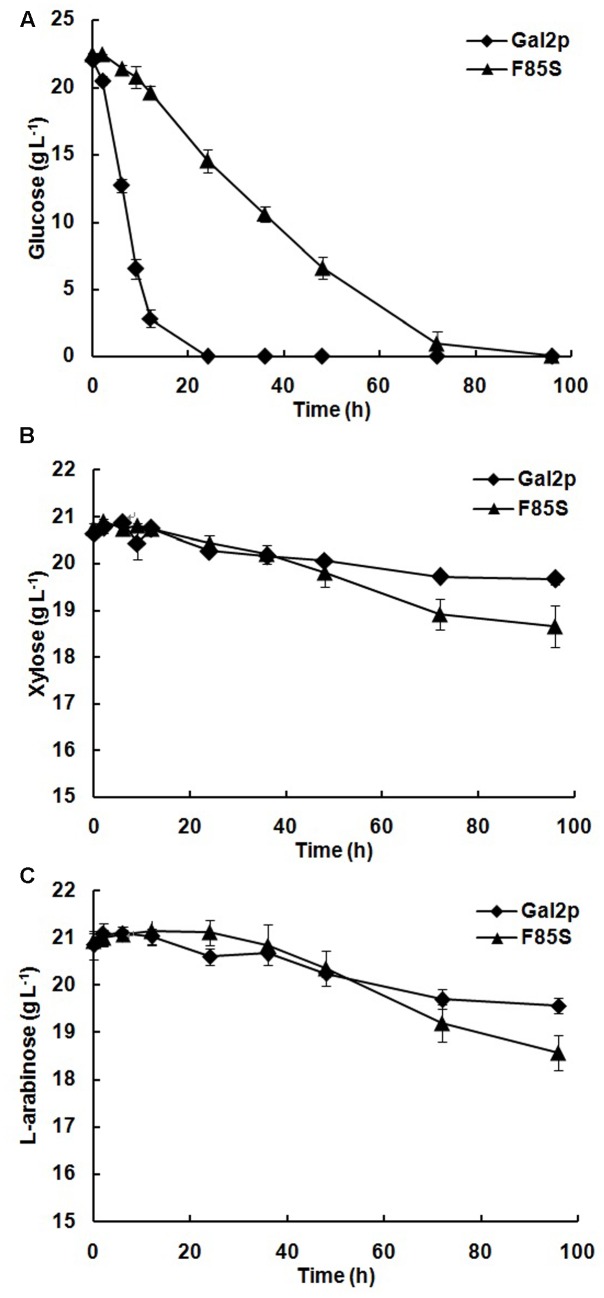
The co-utilization of mutation F85S expressing strain on glucose, xylose, and L-arabinose. Glucose consumption **(A)**, xylose consumption **(B)**, and L-arabinose consumption **(C)** by Gal2p (BSW4EYX-Gal2-A, 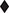) and F85S (BSW4EYX-F85S-A, 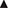). The pre-cultured strains were then cultured in 40 mL SC-LEU-URA medium containing 20 g L^-1^ glucose, 20 g L^-1^ xylose, and 20 g L^-1^ L-arabinose at an initial OD_600_ of 5. The strains were all cultured at 30°C, 200 r min^-1^. The data presented are the averages of three independent tests.

To further test the effect of the best positive mutation F85S on the co-utilization and co-fermentation of glucose, xylose, and L-arabinose, we then transformed the plasmid YIp5-ara (**Table 1**) to the BSW4EYX strains expressing F85S or Gal2p. During a 96 h fermentation for the expressing strains, the mutation F85S decreased the glucose utilization efficiency (**Figure 6A**), obviously increased the xylose consumption amount (**Figure 6B**) by 1.1 g L^-1^, and also increased the L-arabinose consumption amount (**Figure 6C**) by 1 g L^-1^, compared with the original Gal2p. After about 40 h cultivation, the F85S expressing strain could exhibit more significant co-utilization of xylose and L-arabinose with glucose, and the ethanol production was 10 g L^-1^ which was consistent with that of Gal2p.

## Discussion

The utilization of L-arabinose and xylose in lignocellulosic materials is becoming more important for renewable fuel production. The transport efficiency of the two pentoses needs to be increased in the metabolic process of microbial cell factories. Gene knockout and overexpression studies verified the necessary function of Gal2p for L-arabinose transport in *S. cerevisiae* (Wisselink et al., 2010; Wang et al., 2013); therefore, in this study, Gal2p was selected for the identification of amino acid residues that are crucial for L-arabinose transport.

Our group formerly constructed an efficient L-arabinose transport yeast BSW3AP (Wang et al., 2013). After deleting the native Gal2p in this strain, we successfully obtained an L-arabinose transport-deficient chassis, BSW5AP, which is a suitable host cell for L-arabinose transport study. Gal2p is homologous with the xylose permease XylEp of *E. coli*, and so, the outward-facing and partly occluded conformation of XylEp (Sun et al., 2012) was used as the homologous model for L-arabinose binding of Gal2p. A total of nine polar or aromatic amino acid residues were chosen to deeply study the L-arabinose and xylose transport capacity and then seven residues (F85, T89, F223, F350, Y351, N376, and Y446) were shown to significantly affect the L-arabinose transport activity of Gal2p. Meanwhile, compared with directed evolution, site-directed mutagenesis was verified in this study to be a simple and effective method to identify the crucial amino acid residues of pentose transporters.

The F85 position in TMS1 of Gal2p (**Figures 2C,D**) is now verified to have important effect on L-arabinose transport. Changing F85 to polar amino acids S, G, C, T, Y, or N somehow increased the L-arabinose transport activity of Gal2p, otherwise, changing to charged amino acids E or R resulted in a loss of the transport capacity of L-arabinose. This result suggests that the polar chain in this site is useful for L-arabinose binding for processing, but charge forces interferes this process. It is noteworthy that F85S significantly enhanced the exponential growth rates on L-arabinose, and the L-arabinose consumption amounts could be improved by 84%. This mutation also increased the exponential growth rates on xylose by 23%, and consequently decreased the glucose transport preference. The corresponding site of F85 in HXT7p is F79, and the mutant HXT7 (F79S) was previously reported to improve the xylose uptake rate (Reider Apel et al., 2016). Furthermore, F85G not only increased the L-arabinose transport activity, it also decreased preferences for both glucose and xylose transport. This result suggests that the F85G mutant is specific for L-arabinose transport. The F85 site is also present in the conserved motif “GG/FXXXG” in TMS1, which was previously reported to affect the capacity for xylose and glucose transmembrane transport (Young et al., 2014; Knoshaug et al., 2015). This result suggests the conserved role of this site in sugar transport (**Figure 4**).

F223 is another important amino acid residue for L-arabinose transport of Gal2p. When F223 was changed to Q, C, L, and S, the exponential growth rates of the mutants increased, and F223Q significantly improved the exponential growth rate by 16%. Although the F223Q mutation increased the L-arabinose transport capacity, the mutation did not affect xylose or glucose transport. The mutations at residues T89 and N376 significantly decreased the growth of these mutants on L-arabinose, suggesting that these residues are also important for L-arabinose transport. In addition, N376 was formerly shown to affect the xylose transport affinity and preference of Gal2p (Farwick et al., 2014). Random mutation of the F350 and Y351 residues in Gal2p, which are conserved in the motif “YFFYY” and correspond to F334 and F335 in Mgt05196p (Wang et al., 2015), resulted in loss of L-arabinose transport capacity. The phenyl structure of the sites F350 and Y351 might play an important role for L-arabinose exclusion in transport process of Gal2p. Y446 is also highly conserved and necessary for L-arabinose transport of Gal2p, as changes to A, I, C, or S, all resulted in loss of L-arabinose transport capacity. Y446 was also previously reported to be essential for galactose recognition by Gal2p (Kasahara and Kasahara, 2000).

The best mutation F85S of Gal2p was tested to improve the co-utilization of glucose, xylose, and L-arabinose, although the co-fermentation efficiency of xylose and L-arabinose was not high enough and the ethanol production was not significantly increased. F85S partly alleviated the glucose suppression, although the glucose inhibitory effect on xylose and L-arabinose utilization still existed as reported (Subtil and Boles, 2012). Moreover, the chassis cells for testing the sugar co-fermentation of transporter was verified to have low pentose metabolic capacity, but useful for transport link study (Wang et al., 2015). F85S of Gal2p might exhibit more efficient co-utilization of pentose with glucose, in a well engineered diploid *S. cerevisiae* for pentose metabolism.

## Conclusion

In this study, the crucial amino acid residues of Gal2p for L-arabinose transport were predicted and then studied by site-directed mutagenesis. A total of five mutations, F85S, F85G, F85C, F85T, and F223Q, significantly enhanced the L-arabinose transport activity of Gal2p. Meanwhile, F85C, F85S, and F85T mutants also exhibited increased xylose transport capacity. The F85 site was shown to be a key residue in improving the L-arabinose transport activity of Gal2p, especially when changed to polar amino acids. To the best of our knowledge, this is the first study to identify the crucial amino acid residues of the L-arabinose transporter in *S. cerevisiae*. The positive pentose transport mutations, especially F85S of Gal2p, were useful for further improving the utilization efficiency of engineered *S. cerevisiae* for lignocellulosic ethanol production.

## Author Contributions

CW, XB, and YD designed the study. CW, CQ, YL, SW, and JM performed the laboratory work and analyzed the data. CW wrote the manuscript. YD, QZ, YS, and BD advised the manuscript. CW, XB, and YD supported the study.

## Conflict of Interest Statement

The authors declare that the research was conducted in the absence of any commercial or financial relationships that could be construed as a potential conflict of interest.

## Supplementary Material

FIGURE S1 The physical map of plasmid pYX242-*TEF1araA-Gal2Gal2m*.labelFS1
