# Supplementary figures and images for: Identification of Important Amino Acids in Gal2p for Improving the L-arabinose Transport and Metabolism in Saccharomyces cerevisiae
Source: Front Microbiol. 2017 Jul 21;8:1391. doi: 10.3389/fmicb.2017.01391 (PMC5519586; doi:10.3389/fmicb.2017.01391)

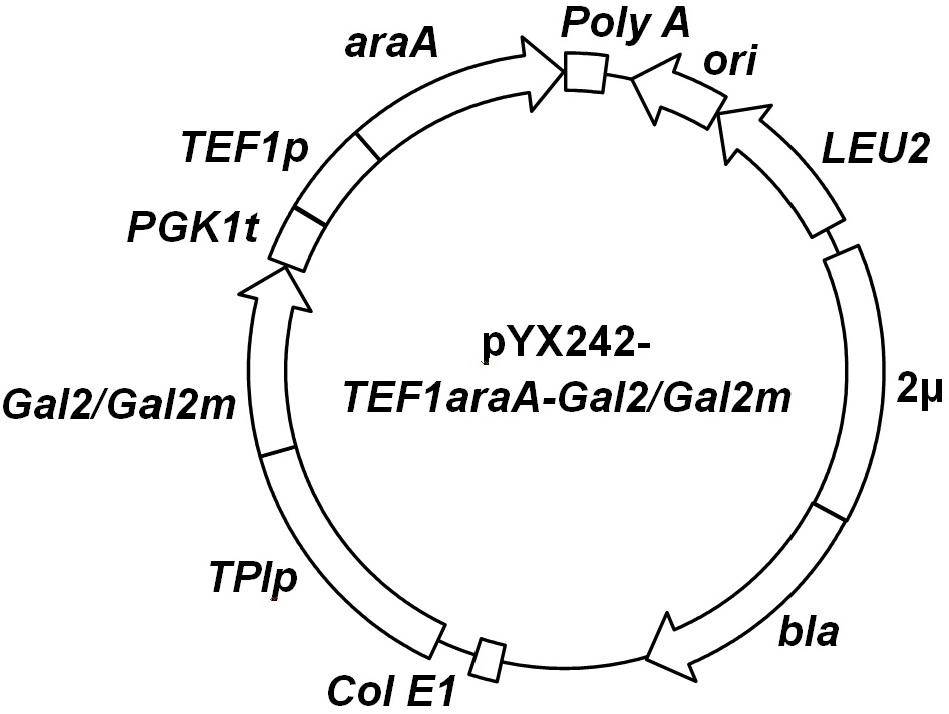

Supplement: FIGURE S1 — The physical map of plasmid pYX242-TEF1araA-Gal2Gal2m.labelFS1 [file Image_1.JPEG]
